# Dental Education

**Published:** 1876-04

**Authors:** 


*Dental Education.*—Dr. S. H. Cartwright, of London, upon this subject remarks :

"The only true reform must consist in higher and more perfect education, and there need be no fear lest dental surgery should not take its position immediately, and a majority of those who practice the specialty become fully qualified practitioners. The manufacture of mechanical substitutes for the natural dentures, I would gladly relegate to a body of skilled mechanicians who should hold a similar position to that of the ordinary manufacturer of surgical appliances; the instruments being made and adjusted, subject to the supervision of a surgeon who, especially in dental practice, should have a competent knowledge of mechanics, as a mere machinist might fail in that æsthetic, artistic and anatomical perception which is necessary in cases of deformity like those alluded to here.

But this division will not achieve all that which is to be desired ; for those who are ambitious for a higher professional and social position must enter their profession a portal, the entrance to which should imply a liberal education, both general and special; and this is to be found in the diploma of a member of the Royal College of Surgeons, to which it is to be regretted, that a special degree in dental surgery was not formerly appended. One of our most distinguished and celebrated surgeons lately said, while speaking authoritatively in the name of the Royal College of Surgeons, that that body would be only too glad to receive the best men of that specialty into his fold.

I trust that ere long, every general hospital will have a special department for instructions in dental diseases, as for those of the eye, throat or ear.

I am certain that the attempt to separate the dental profession of surgery from that of general surgery would be a great mistake. In America such a mistake has been made; and we find that the children are now seeking a connection which the parent justly disclaims, save on the condition that they educate taemselves as fully qualified practitioners.

Finally, if specialists are ambitious of the status granted to their medical confreres, they have only to educate themselves as these are educated. Let them dictate a large and more liberal view of their calling, banish all intestine bickering. Let them advance schemes for the common weal apart from the mere personal interests and the gratification of personal ambition. Men who now hold aloof from special fields, and the general body of those who practice the specialty adopted, would then join the van of those who elevate the good name and support the fair fame of their profession in word and action."

---

